# Efficacy of transcatheter arterial chemoembolization for liver metastases arising from pancreatic cancer

**DOI:** 10.18632/oncotarget.14642

**Published:** 2017-01-13

**Authors:** Jun-Hui Sun, Tan-Yang Zhou, Yue-Lin Zhang, Guan-Hui Zhou, Chun-Hui Nie, Tong-Yin Zhu, Sheng-Qun Chen, Bao-Quan Wang, Song Ye, Yan Shen, Hua Guo, Wei-Lin Wang, Shu-Sen Zheng

**Affiliations:** ^1^ Hepatobiliary and Pancreatic Interventional Treatment Center, Division of Hepatobiliary and Pancreatic Surgery, China; ^2^ Key Laboratory of Precision Diagnosis and Treatment for Hepatobiliary and Pancreatic Tumor of Zhejiang Province, China; ^3^ Key Laboratory of Combined Multi-organ Transplantation, Ministry of Public Health, Zhejiang, Hangzhou, China; ^4^ Collaborative Innovation Center for Diagnosis Treatment of Infectious Diseases, Zhejiang University, Hangzhou, Zhejiang, China

**Keywords:** pancreatic tumor, hepatic metastasis, TACE, intervention, efficacy

## Abstract

**Purpose:**

The aim of the study was to evaluate the efficacy of transcatheter arterial chemoembolization (TACE) in treating patients with liver metastases from pancreatic cancer, and explore the prognostic risk factors.

**Results:**

Three of the 27 patients were totally recovered, and 12 were partially alleviated. The total efficacy rate was 55.6% (15/27). The median survival time was 13.6 months, and the 0.5-, 1-, 2-, 3-, and 5-year survival rates were 70.4% (19/27), 48.1% (13/27), 22.2% (6/27), 14.8 (4/27), 11.1% (3/27), respectively. None of the groups showed any severe complications. Univariate analysis showed that pathological type, concomitant therapies for liver metastasis, vascular supply, CA199 levels and extrahepatic metastasis were related to prognosis (P < 0.05). Multivariate analysis indicated that pancreatic cancer pathology and extrahepatic metastasis were independent risk factors influencing patients' prognosis (χ2 = 13.182, 17.989, *P* < 0.05).

**Methods:**

The clinical records of 27 patients with lliver metastases from pancreatic cancer diagnosed at the First Affiliated Hospital of Zhejiang University between May 2009 and May 2015 were retrospectively analyzed. The short-term and long-term efficacy and toxic side effects of TACE were observed. The prognostic risk factors were analyzed using Cox (proportional hazards) regression model.

**Conclusion:**

TACE is an effective therapy for treating liver metastases from pancreatic malignancy. Pathological type and extrahepatic metastasis of pancreatic tumor are independent risk factors for patients' prognosis. The prognosis of patients with liver metastasis from pancreatic neuroendocrine neoplasm is superior to that of extrahepatic metastasis.

## INTRODUCTION

Pancreatic malignancy ranks tenth or eleventh among all malignancies in China [1]. Surgical resection is the only radical cure [2–4]. However, most patients manifest multiple liver metastases at the time of diagnosis; liver metastasis also occurs postoperatively in some patients. The overall survival (OS) for patients with liver metastases from pancreatic cancer is only 11.1 months [4]. Clinically, chemoradiotherapy is recommended for pancreatic cancer metastases. Gemcitabine remains the backbone of the standard of care for these patients and results in a median OS of 5–7 months. Performance status, tumor and serum markers, disease burden, and metastatic pattern have also been described as prognostic and predictive factors for survival in pancreatic cancer patients[5]. Recent technological advances suggest that micro-traumatic interventions, such as transcatheter arterial chemoembolization (TACE), are clinically effective for the treatment of liver metastases arising from pancreatic malignancy[5–6]. We retrospectively analyzed the early-stage efficacy of TACE in 27 patients with pancreatic liver metastases, and explored the related prognostic factors.

## RESULTS

### Efficacy of TACE

The 27 patients underwent TACE for a total of 52 times (1–7 times each), including eight treatments with drug-eluting beads (DEB) and 44 treatments with iodinated oil. The time from diagnosis of liver metastasis to the first TACE intervention was 1~10 months, with an average of 4.5 months. The first TACE therapy included 13 cases of single liver metastasis and 14 cases of liver metastases combined with extrahepatic lesions (pancreatic cancer primary focus or relapse; lymphatic metastasis; or colon, spleen, or adrenal metastasis). Before TACE, three patients underwent partial liver excision and 12 received systemic intravenous chemotherapy (1~10 courses); four of these had combined RFA and HIFU therapy. The remaining 12 cases received no special treatments.

### Complications and adverse events

There were no serious local side effects observed over the treatment course. After treatment was completed, anorexia (grade 1 according to the Common Terminology/Criteria for Adverse Events [CTCAE][7]) occurred in 20 patients, bone marrow suppression (grade 1) occurred in three patients, and epigastric pain (grade 1) occurred in 23 patients. All of these complications were significantly relieved with symptomatic treatment.

### Imaging evaluations

**Figure 1:**
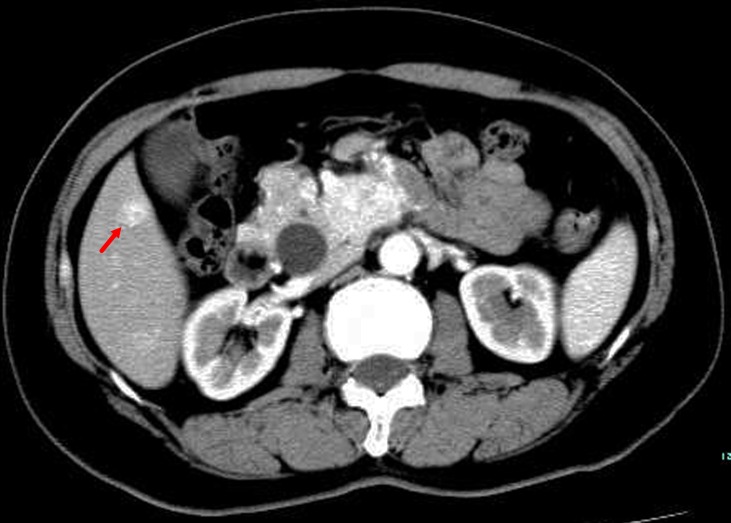

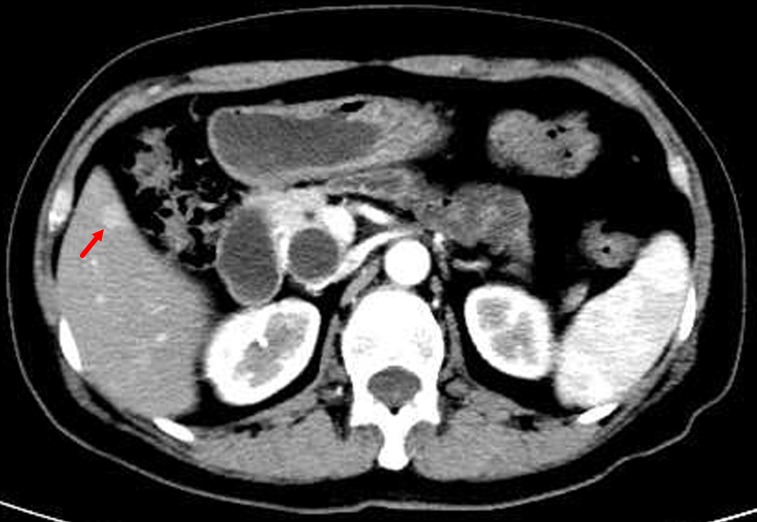
TACE in a 59-year-old female with multiple liver metastases form pancreatic neuroendocrine tumor **A**. Contrast-enhanced CT before TACE revealed a segment 6 hypovascular liver metastasis in the arterial phase (arrow) **B**. contrast-enhanced CT showed a significant decrease in the lesion at 6 months after treatment (arrow).

**Figure 2:**
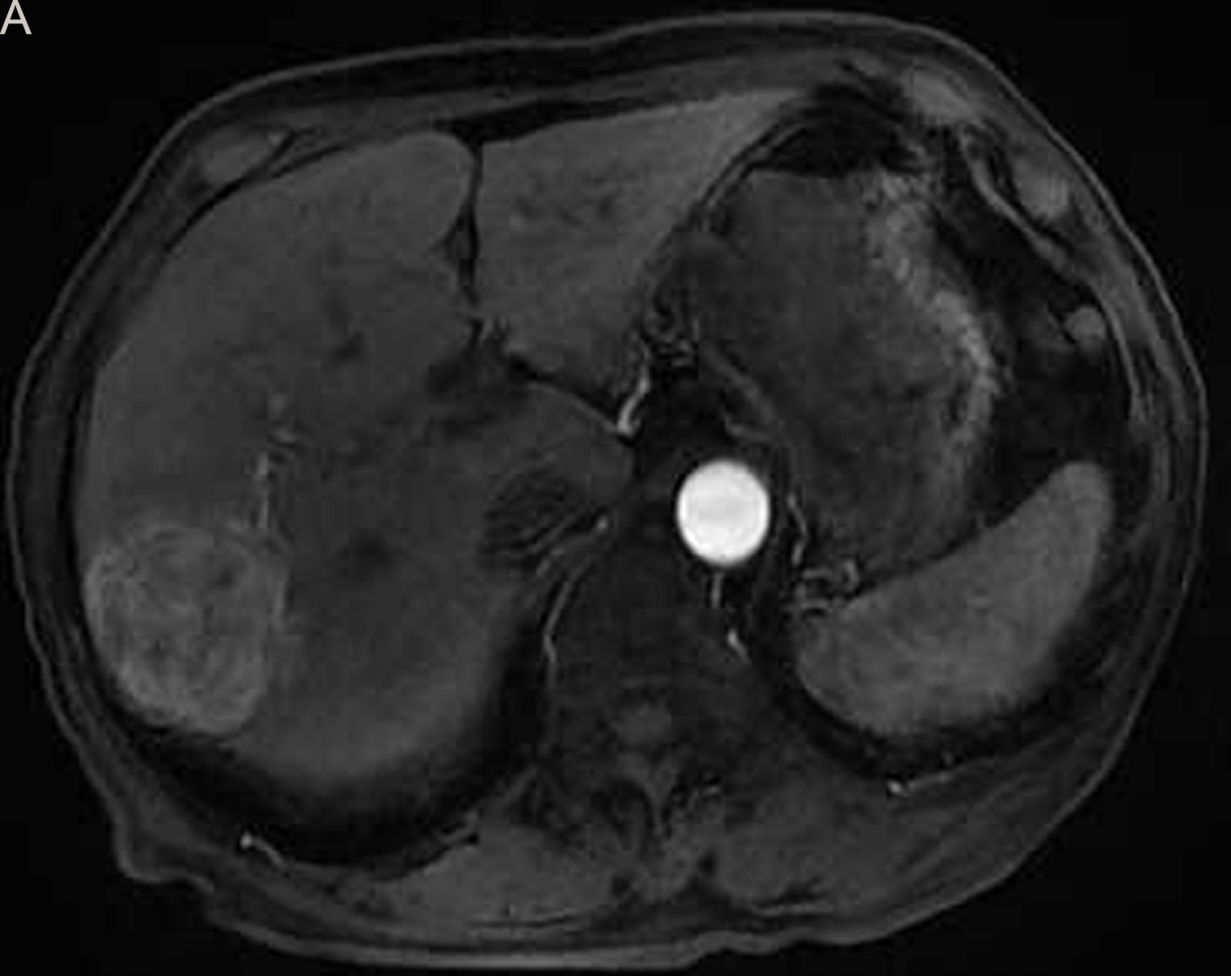

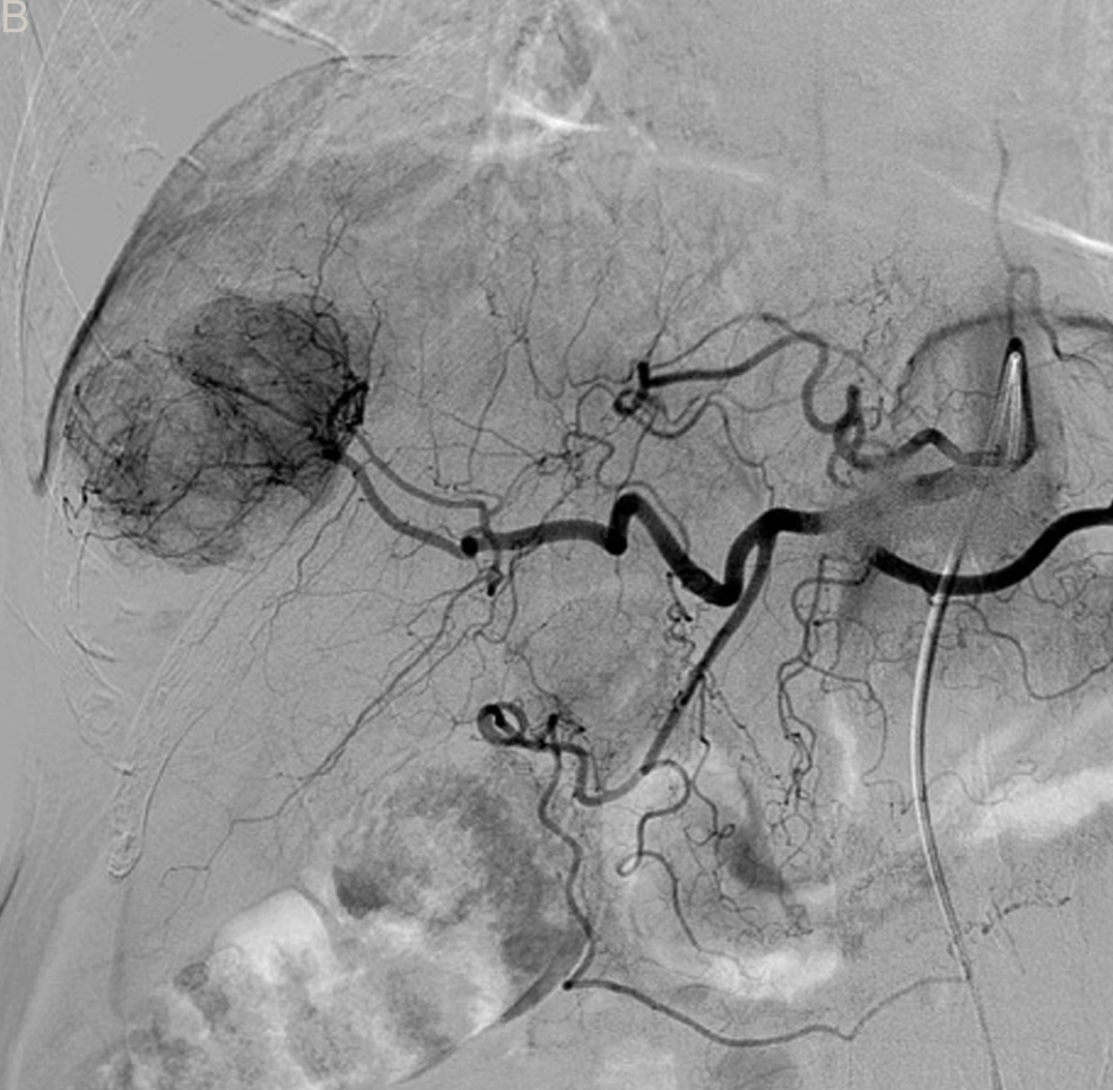

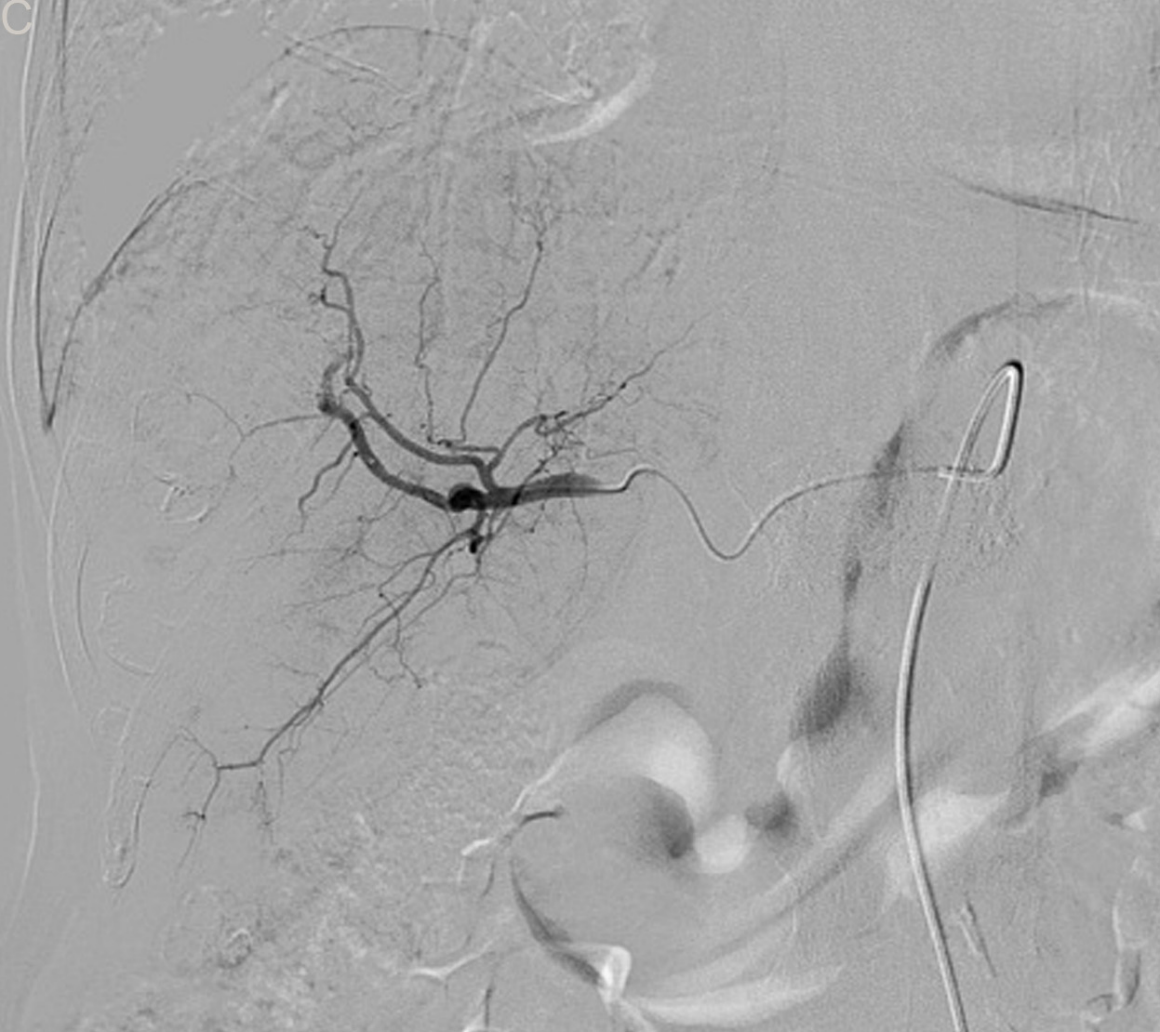

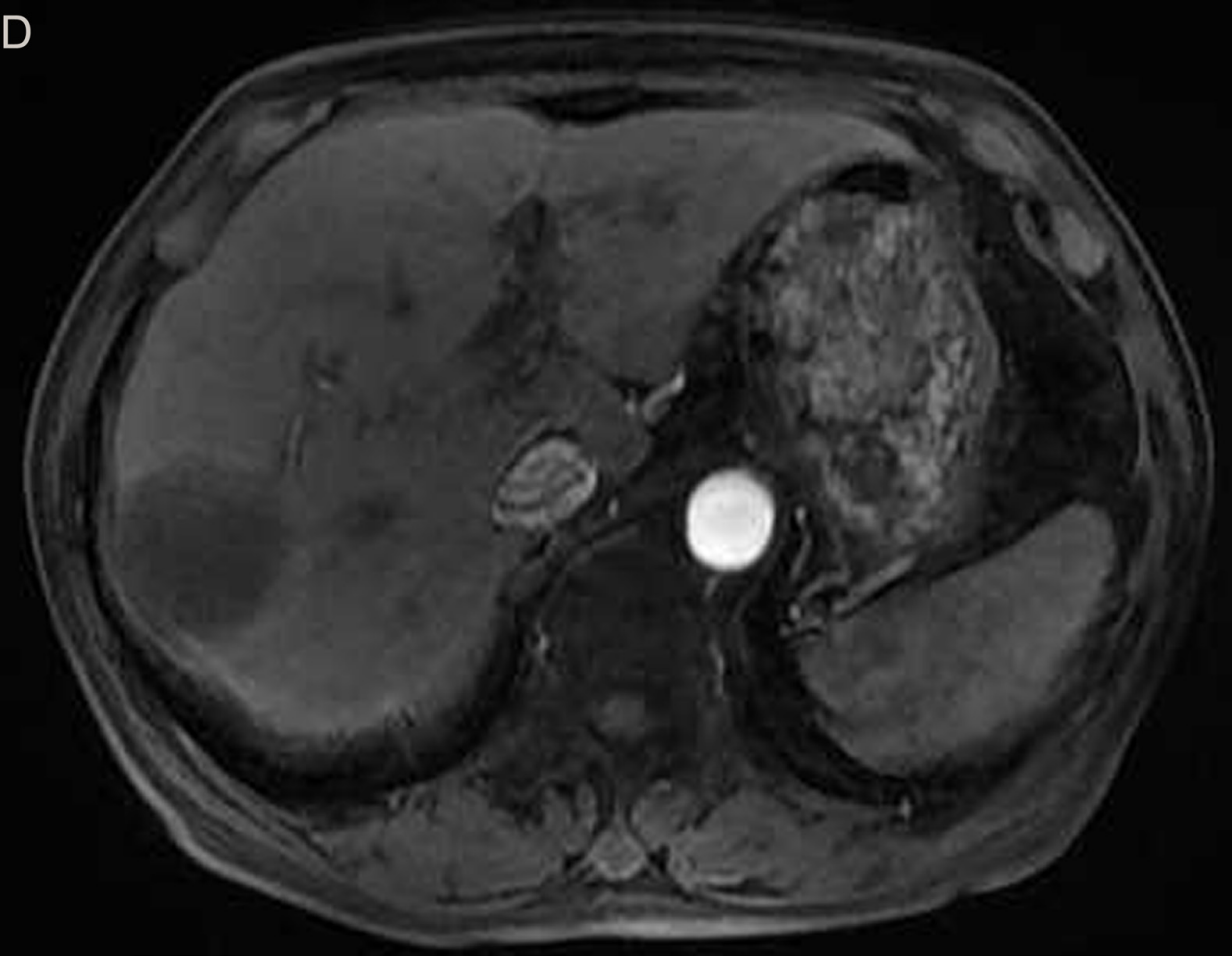
Eighty six-year-old male with a huge hypervascular metastatic pancreatic neuroendocrine tumor treated with multiple cycles of chemotherapy **A**. Gd-enhanced T1WI revealed a round enhanced tumor of the right lobe in the arterial phase. **B**. Tumor staining during arterial phase of DSA: The tumor was nourished by the branches of the right hepatic artery. **C**. Tumor staining disappeared after TACE therapy. **D**. Gd-enhanced T1WI 2 image months after drug-eluting microspheres loaded with oxaliplatin chemoembolization showed signal reduction indicating resorption without enhancing residual or recurrent tumor mass.

According to the imaging findings from before and after therapy, excluding the one case that was lost to follow-up, there were 11 PR cases (42.3%), 10 SD cases (38.5%), 5 PD cases (19.2%), and no CR cases. The efficacy rate was 42.3% (Figures 1 and 2).

### Survival analysis

**Figure 3:**
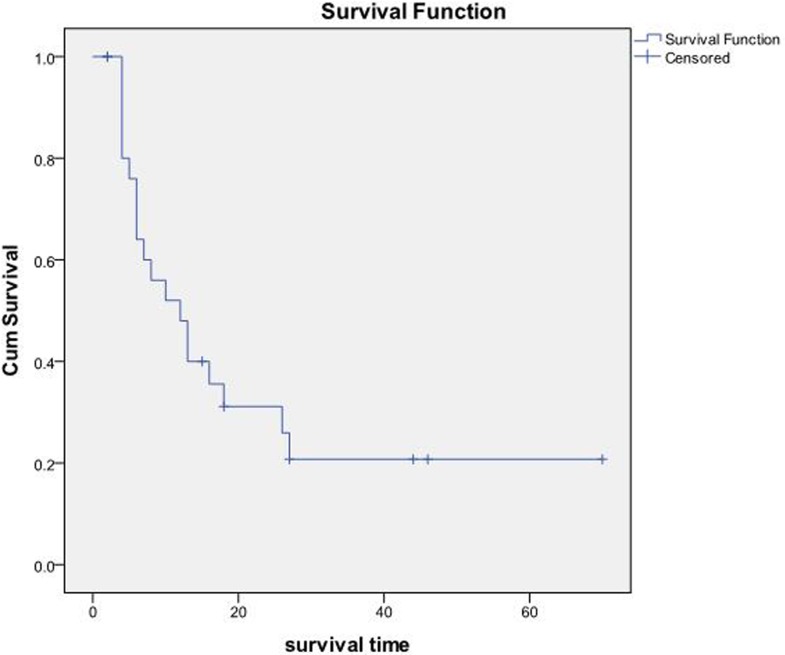

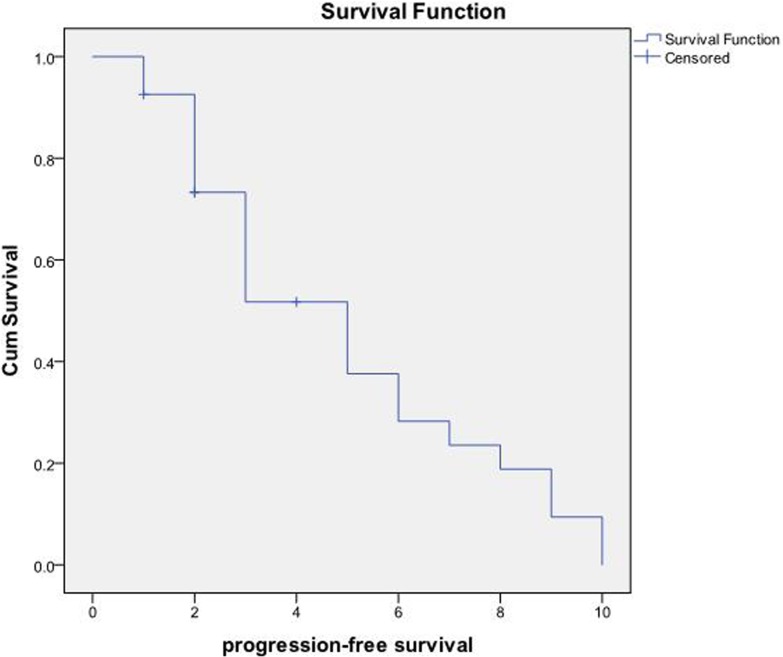
**A**. The OS and median OS of the 27 patients were (23.02±5.18) months and (12.00±3.12) months, respectively. **B**. PFS and the median PFS were (4.87+0.60) months and (5.00±0.85) months, respectively.

**Figure 4:**
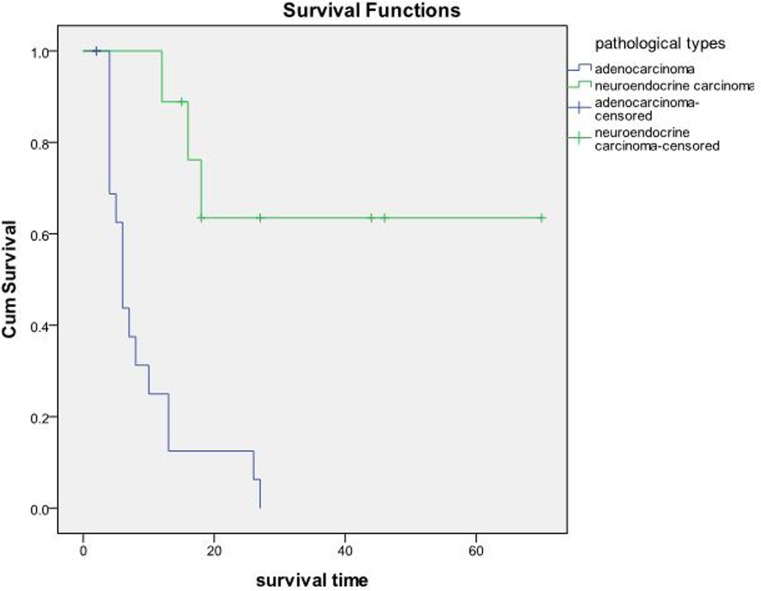

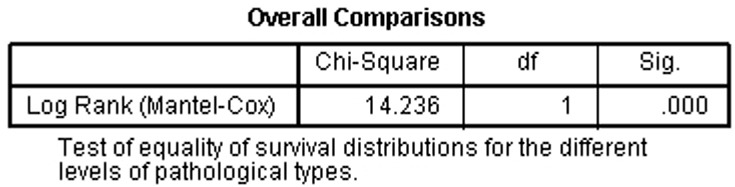
18 cases in the pancreatic cancer group with an OS of 9.19±1.85 months, and nine cases in the pancreatic neuroendocrine tumor group with an OS of 50.10±9.21 months *P* < 0.05

The OS and PFS of the 27 patients were 23.02±5.18 months and 4.87±0.60 months, respectively (Figure 3). According to the original pathological type, 18 patients in the pancreatic cancer group showed an OS of 9.19±1.85 months, and nine cases in the pancreatic neuroendocrine tumor group showed an OS of 50.10±9.21 months (Figure 4). Based on the combination of intrahepatic metastatic foci with other therapies, 12 cases undergoing single TACE therapy showed an OS of 7.91±2.10 months, and 15 cases in the combination group exhibited an OS of 35.53±7.81 months. Based on extrahepatic metastases, 13 patients were diagnosed with more than one extrahepatic metastasis. These included metastases to the spleen, lymph nodes, adrenal glands, and other organs, with an OS of 10.75±2.38 months. The 14 cases that showed no other metastases outside the liver had an OS of 31.72±8.21 months. There was a statistical significance between the two groups (P<0.05). Based on the primary surgical focus, there were 12 excision cases with an OS of 32.36±8.56 months, and 15 non-surgical cases of excision with an OS of 11.21±2.45 months.

### Prognostic risk factors

#### Univariate analysis

Pathology type, combination therapy, vascular supply, CA19-9 levels, and extrahepatic metastases were significantly correlated with prognosis (P<0.05).

#### Multivariate analysis

**Figure 5:**
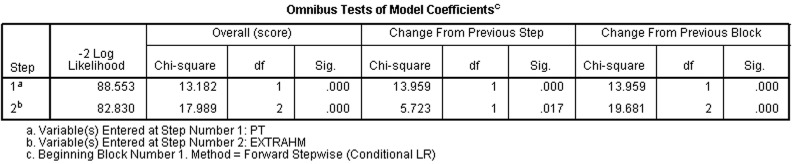
Patients' pancreatic tumor pathology and extrahepatic metastasis were significantly correlated with prognosis (χ2=13.182, 17.989 *P* < 0.05)

Pancreatic tumor pathology and extrahepatic metastases were significantly correlated with prognosis (χ2=13.182, 17.989; P<0.05) (Figure 5).

## DISCUSSION

Malignant pancreatic tumors are very common clinically, and surgical resection is the only radical curative method[8]. However, only a few patients undergo surgery, and most of these are diagnosed with liver metastasis. The incidence of liver metastasis is the most important factor influencing prognosis[9]. Therefore, effective therapy for liver metastasis is critical to ensure optimal palliative management[10]. Surgery or radiofrequency ablation are recommended for patients with solitary liver metastases from pancreatic cancer. The treatment limitations include multiple disabilities, high tumor load, and technically challenging liver metastases.

Recently, TACE for liver metastases via the hepatic artery was reported to be clinically efficacious[11–12]. Similar to primary liver cancer, the main blood vessel that supplies liver metastases is also a branch of the hepatic artery. Thus, selective TACE according to the characteristic distribution of the tumor blood vessels is an important method for the local treatment of liver metastases originating from pancreatic cancer[13]. Brown et al.[14] reported remission in a single patient with pancreatic liver metastases after chemoembolization based on gemcitabine/cisplatin, which was confirmed by imaging, leading to an OS of up to one year. Homma et al.[15] blocked part of the blood flow in the pancreas by super-selective catheterization, and altered the hemodynamics in the pancreas. The catheter was inserted into the splenic artery and/or the hepatic artery, and the other end was connected to a chemotherapy pump for continuous-infusion chemotherapy. A total of 16 patients diagnosed with liver metastases from pancreatic cancer underwent therapy, with a response rate of 68.8% and OS of 16.3 months. The study found that reconstruction of the blood supply between the pancreatic cancer and the liver metastases increased chemotherapy drug concentrations in the local blood circulation, effectively controlling the disease and prolonging survival. Azizi et al.[16] reported 32 patients with liver metastases from pancreatic malignancy treated with multiple TACE (gemcitabine, cisplatin, and mitomycin). The OS was up to 16 months and PFS was 6 months, suggesting that repeated TACE controlled tumor foci and resulted in long-term benefit. The number of metastatic liver foci did not affect TACE efficacy. In our study, we used TACE to treat 27 patients with liver metastases arising from the pancreas, with an OS up to 23 months. The OS was nine months in the pancreatic cancer group and 50 months in the pancreatic neuroendocrine tumor group. The differences between previous studies and the present one may be due to the fact that the patients in our study were at a more advanced clinical stage, and most of them had extrahepatic metastases. Moreover, most of the patients in our study only received a single TACE session.

Currently, some of the embolic agents used in TACE include ultra-fluid lipiodol, gelatin sponge granules, PVA particles, and DEB. Recently in China, DEB became widely used, providing a new regimen for TACE therapy for hepatic metastasis[17]. Due to the small sample size, DEB was combined with adriamycin, irinotecan, and oxaliplatin. After catheter-based administration into the artery supplying the tumor, the end of the artery is reached during the blood flow, forming emboli on the tumor vessels. After embolization, the drug is continuously released at the tumor site, resulting in increased clinical efficacy against poorly vascularized liver metastases[18]. Compared with traditional TACE, DEB-TACE facilitates sustained local drug delivery, eliciting a higher response rate and a lower incidence of adverse reactions. Kotoyan et al.[19] used DEB-TACE to treat 10 patients with pancreatic liver metastases, including six with adenocarcinoma and four with neuroendocrine neoplasms. These ten patients received 17 sessions of DEB-TACE (one to three per patient). The six adenocarcinoma patients were given DEB with irinotecan, while the four neuroendocrine tumor patients were treated with DEB conjugated with adriamycin. The efficacy rates after six and 12 months were 80% and 75%, respectively, and the median OS was 9.3 months. In the present study, 27 patients underwent TACE for a total of 52 times (1~7 times per patient). Eight of the 27 patients received DEB loaded with adriamycin, oxaliplatin, and irinotecan. Seven neuroendocrine neoplasm patients manifested an OS of 8~63 months. Therefore, we believe that in patients with liver metastases due to pancreatic cancer, especially those with poorly vascularized metastatic tumors, DEB was clinically efficacious and should be recommended as an alternative to chemoradiotherapy for liver metastases arising from pancreatic cancer.

There are multiple risk factors influencing the OS of patients with pancreatic tumor metastases. Zanini et al[20]performed resection of metastases in 15 patients with solitary liver metastases arising from the pancreas and analyzed the potential prognostic risk factors. The results indicated that the only potential prognostic risk factor was the presence of liver metastasis (i.e., pancreatic cancer accompanied by liver metastasis during therapy or liver metastasis manifesting after surgery). Furthermore, the prognosis of pancreatic cancer patients with liver metastasis after surgery was better than that associated with liver metastasis during therapy, with median OS of 11.4 months and 8.3 months, respectively. Bertani et al[21] performed a multivariate analysis of 43 patients with pancreatic neuroendocrine tumors presenting with liver metastases, with tumors that were not completely excised. The results showed that pancreatic primary cancer excision, age, Ki-67 level, and liver tumor load were the main factors influencing prognosis. A multivariate analysis of the 27 patients in our study demonstrated that the pathological type and metastasis were the two key prognostic factors. The prognosis for pancreatic neuroendocrine tumor with metastatic foci limited to the liver was better than that for pancreatic liver metastases and malignant tumors associated with extrahepatic metastases. The multivariate analysis did not show a role for primary tumor excision in the prognosis, which could be related to the small sample size. Thus, larger sample sizes should be used in future studies to arrive at more robust conclusions.

Overall, the outlook for pancreatic liver-associated metastases is still poor, with a low possibility for cure. The goal of therapy is to increase the OS of patients and to improve disease-related symptoms and quality of life. TACE therapy improves symptoms and quality of life; therefore, we believe that TACE for pancreatic cancer-related liver metastasis is an effective local therapy. The tumor type and metastatic condition are the key factors influencing OS of these patients after therapy.

## MATERIALS AND METHODS

### Patients

The clinical records of 27 patients diagnosed with liver metastases from pancreatic cancer and who were treated with TACE between May 2009 and May 2015 were retrospectively reviewed. The 27 patients included 17 males and 10 females, aged between 39 and 86 years, with a median age of 59 years. There were 19 cases with a primary focus in the pancreatic body and tail, and eight involving other sites. The 27 patients included 15 with CA19-9 positivity, 12 with negative CA19-9, 12 with a single liver metastasis, and 15 with metastases to the liver, lymph nodes, adrenal glands, spleen, and colon. Nine patients were surgically diagnosed with pancreatic neuroendocrine tumors or with liver metastases using pathological tests combined with immunohistochemistry. Ten patients had histologic and radiologic proof of metastatic pancreatic cancer to the liver based on percutaneous biopsy. Another eight cases were staged by our two senior imaging experts.

### Therapy

All of the patients signed informed consent. Before TACE, each patient underwent routine blood and urine examinations for hepatorenal and coagulation function, in addition to enhanced CT or MRI of the liver. A 5F RH catheter (Cook Inc., Bloomington, IN, USA) or a 5F Yashio catheter (Terumo Co., Tokyo, Japan) was inserted via the femoral artery to perform selective superior mesenteric artery radiography, and to identify the shape and unobstructed status of the hepatic artery and portal vein for superselective hepatic artery angiography. According to the number of intrahepatic metastatic foci, their locations, and the blood supply, TACE was performed. The chemotherapy drugs included pirarubicin hydrochloride (10 mg/bottle, Shenzhen Main Luck Pharmaceuticals Inc., Shenzhen, China) at a dose of 30 mg mixed with lipiodol (Lipiodol®, Laboratoire Guerbet, Aulny-sous-bois, France), or at 80 mg for DEB-TACE treatment. Gemcitabine 1000 mg (Jiangsu Haosen Medicine Co., Ltd., Lianyungang, China), oxaliplatin 150 mg (Jiangsu Hengrui Medicine Co., Ltd.), and irinotecan 80 mg (Jiangsu Hengrui Medicine Co., Ltd.) were also used for perfusion chemotherapy. The perfusion chemotherapy drugs were administered according to the general condition of the patient and the intrahepatic metastatic foci, and included single drugs, doublets, or triplets. Lipiodol and/or PVA particles (350–510 μm, Ailikang Medicine Co., Ltd, Hangzhou, China) and Embosphere® (Embospheres, Biosphere Medical, Rockland, MA, USA) were used as embolization materials. The degree of embolism was controlled by significantly decreasing the cerebral artery flow velocity, and the tumor staining disappeared. All patients received supportive treatments after the TACE procedure, including antibiotic prophylaxis, liver protection, antacid agents, and antiemetics. Follow-up laboratory tests, including blood cell counts and liver function parameters, were routinely performed at one-month intervals. Follow-up imaging was performed in all patients 4–6 weeks after each TACE treatment. The TACE treatments were repeated if a new or residual tumor was detected on follow-up imaging.

### Therapeutic effect

Changes in imaging and OS, as well as progression-free survival (PFS), were used as indicators of efficacy of the treatment against liver metastasis. All of the patients underwent enhanced CT or MRI before the operation. During follow-up at 4–6 weeks after the first interventional therapy, all of the abovementioned tests were conducted, then repeated in the next 1–6 months based on the therapeutic outcome. The treatment response was evaluated 4–6 weeks after each TACE treatment using the modified Response Evaluation Criteria in Solid Tumors (mRECIST)[22]. A complete response (CR) was defined as disappearance of any intratumoral arterial enhancement in all lesions; a partial response (PR) was defined as a 30% decrease in the sum of the diameters of viable (contrast enhancement in the arterial phase) lesions; progressive disease (PD) was defined as an increase of 20% in the sum of the diameters of viable lesions; and stable disease (SD) was defined as any case that did not qualify as either PR or PD. The two largest foci were selected among the multifocal lesions for measurement. CR and PR were considered as valid. OS refers to the time starting at initial interventional therapy until death or final follow-up (May 2016). PFS is the time since the first interventional therapy until the development of intrahepatic foci.

### Follow-up

The follow-up visits were conducted via telephone or outpatient services. The end of follow-up was defined by the elapsed time between initial interventional therapy until death or the final follow-up (May 2016), which ranged from 3 to 70 months (median 13 months). One case was lost to follow-up 6 months after treatment, for an overall follow-up rate of 96.3% (26/27).

### Statistical analysis

All statistical analyses were performed using SPSS version 19.0 software (IBM SPSS Statistics, IBM Corporation, Armonk, NY, US). Kaplan-Meier survival curves were used to analyze OS and PFS, and to calculate the total survival rate. Univariate analysis of prognostic factors was tested by log-rank, and Cox regression was used in the multivariate analysis. P<0.05 was deemed statistically significant.
