# Correction: Efficacy of transcatheter arterial chemoembolization for liver metastases arising from pancreatic cancer

**DOI:** 10.18632/oncotarget.24759

**Published:** 2018-03-16

**Authors:** Jun-Hui Sun, Tan-Yang Zhou, Yue-Lin Zhang, Guan-Hui Zhou, Chun-Hui Nie, Tong-Yin Zhu, Sheng-Qun Chen, Bao-Quan Wang, Song Ye, Yan Shen, Hua Guo, Wei-Lin Wang, Shu-Sen Zheng

**Affiliations:** ^1^ Hepatobiliary and Pancreatic Interventional Treatment Center, Division of Hepatobiliary and Pancreatic Surgery, First Affiliated Hospital, College of Medicine, Zhejiang University, China; ^2^ Key Laboratory of Precision Diagnosis and Treatment for Hepatobiliary and Pancreatic Tumor of Zhejiang Province, China; ^3^ Key Laboratory of Combined Multi-organ Transplantation, Ministry of Public Health, Zhejiang, Hangzhou, China; ^4^ Collaborative Innovation Center for Diagnosis Treatment of Infectious Diseases, Zhejiang University, Hangzhou, Zhejiang, China

**This article has been corrected:** The proper name of the institution is as follows:

^1^ Hepatobiliary and Pancreatic Interventional Treatment Center, Division of Hepatobiliary and Pancreatic Surgery, First Affiliated Hospital, College of Medicine, Zhejiang University, China

Original article: Oncotarget. 2017; 8:39746-39755. https://doi.org/10.18632/oncotarget.14642

